# Activation of Wnt/*β*-catenin signalling pathway induces chemoresistance to interferon-*α*/5-fluorouracil combination therapy for hepatocellular carcinoma

**DOI:** 10.1038/sj.bjc.6605064

**Published:** 2009-04-28

**Authors:** T Noda, H Nagano, I Takemasa, S Yoshioka, M Murakami, H Wada, S Kobayashi, S Marubashi, Y Takeda, K Dono, K Umeshita, N Matsuura, K Matsubara, Y Doki, M Mori, M Monden

**Affiliations:** 1 Department of Surgery, Graduate School of Medicine and Health Science, Osaka University, Osaka, Japan; 2 Department of Health Science, Graduate School of Medicine and Health Science, Osaka University, Osaka, Japan; 3 Department of Molecular Pathology, Graduate School of Medicine and Health Science, Osaka University, Osaka, Japan; 4 DNA Chip Research Inc., Kanagawa, Japan

**Keywords:** hepatocellular carcinoma, combination therapy, interferon-*α*, 5-fluorouracil, chemoresistance, Wnt signalling

## Abstract

Type I IFN receptor type 2 (IFNAR2) expression correlates significantly with clinical response to interferon (IFN)-*α*/5-fluorouracil (5-FU) combination therapy for hepatocellular carcinoma (HCC). However, some IFNAR2-positive patients show no response to the therapy. This result suggests the possibility of other factors, which would be responsible for resistance to IFN-*α*/5-FU therapy. The aim of this study was to examine the mechanism of anti-proliferative effects of IFN-*α*/5-FU therapy and search for a biological marker of chemoresistance to such therapy. Gene expression profiling and molecular network analysis were used in the analysis of non-responders and responders with IFNAR2-positive HCC. The Wnt/*β*-catenin signalling pathway contributed to resistance to IFN-*α*/5-FU therapy. Immunohistochemical analysis showed positive epithelial cell adhesion molecule (Ep-CAM) expression, the target molecule of Wnt/*β*-catenin signalling, only in non-responders. *In vitro* studies showed that activation of Wnt/*β*-catenin signalling by glycogen synthesis kinase-3 inhibitor (6-bromoindirubin-3′-oxime (BIO)) induced chemoresistance to IFN-*α*/5-FU. BrdU-based cell proliferation ELISA and cell cycle analysis showed that concurrent addition of BIO and IFN-*α*/5-FU significantly to hepatoma cell cultures reduced the inhibitory effects of the latter two on DNA synthesis and accumulation of cells in the S-phase. The results indicate that activation of Wnt/*β*-catenin signalling pathway induces chemoresistance to IFN-*α*/5-FU therapy and suggest that Ep-CAM is a potentially useful marker for resistance to such therapy, especially in IFNAR2-positive cases.

Interferon (IFN) is a regulatory cytokine with various cellular activities, such as anti-proliferative, immunomodulatory and anti-angiogenic activities (Baron and Dianzani, 1994; Gutterman, 1994). Several studies emphasised the strong anti-tumour activity of IFN in hepatocellular carcinoma (HCC), when used in combination with other chemotherapeutic agents (Patt *et al*, 1993; Obi *et al*, 2006). We also reported the clinical efficacy of IFN-*α*/5-fluorouracil (5-FU) combination therapy for advanced HCC (Miyamoto *et al*, 2000; Sakon *et al*, 2002; Ota *et al*, 2005; Nagano *et al*, 2007a, 2007b) and the mechanism of its anti-tumour effects (Eguchi *et al*, 2000; Yamamoto *et al*, 2004; Kondo *et al*, 2005; Nakamura *et al*, 2007; Wada *et al*, 2007). Further studies showed that the expression of IFN receptor type 2 (IFNAR2) in HCC tissue samples correlates significantly with clinical response to IFN-*α*/5-FU combination therapy (Ota *et al*, 2005; Nagano *et al*, 2007a). In an earlier study, we reported that 66% of those who responded to such treatment were IFNAR2-positive, but half of these 20 patients showed no clinical response (Nagano *et al*, 2007a). Therefore, finding novel biological markers of resistance to IFN-*α*/5-FU combination therapy is desirable, not only so that non-responders receive other potentially more successful treatments, but also to avoid their suffering caused by debilitating side effects.

Development of microarray technology has facilitated analysis of genome-wide expression profiles (Zembutsu *et al*, 2002; Yang *et al*, 2005). It can generate a large body of information concerning genetic networks related to pathological subtype, prognosis and resistance to anticancer drugs of neoplasm. We have reported many studies using PCR array or oligonucleotide microarray system in various human malignancies, particularly in gastrointestinal and HCCs (Komori *et al*, 2008; Kurokawa *et al*, 2004a, 2004b; Motoori *et al*, 2005, 2006). To understand the complex biological processes, such as cancer progression and drug resistance, it is also important to consider differential gene expression by the gene network analysis (Kittaka *et al*, 2008). A detailed human interactive network that captures the entire cellular network would be invaluable in interpreting cancer signatures (Calvano *et al*, 2005; Rhodes and Chinnaiyan, 2005).

In this study, we applied the methods of oligonucleotide microarray system and gene network analysis to identify informative gene set(s) and signalling pathway(s) related to resistance to IFN-*α*/5-FU combination therapy, especially in patients with IFNAR2-positive HCC. The results showed that Wnt/*β*-catenin signalling influenced resistance to IFN-*α*/5-FU combination therapy. The study also investigated the potential importance of epithelial cell adhesion molecule (Ep-CAM), which is encoded by the TACSTD1 gene and confirmed as one of the target genes of Wnt/*β*-catenin signalling (Yamashita *et al*, 2007), as a biological marker for resistance to IFN-*α*/5-FU combination therapy.

## Materials and methods

### Cell lines

The human HCC cell lines, PLC/PRF/5, HuH7, HLE, HLF and HepG2, were purchased from the Japanese Cancer Research Resources Bank (Tokyo, Japan). The Hep3B cell line was obtained from the Institute of Development, Aging and Cancer, Tohoku University (Sendai, Japan). They were maintained in Dulbecco's Modified Eagle Medium supplemented with 10% foetal bovine serum, 100 U ml^−1^ penicillin and 100 *μ*g ml^−1^ streptomycin at 37°C in a humidified incubator with 5% CO_2_ in air.

### Drugs and reagents

Purified human IFN-*α* was kindly supplied by Otsuka Pharmaceutical Co. (Tokyo, Japan) and 5-FU was obtained from Kyowa Hakko Co. (Tokyo, Japan). The small molecule of 6-bromoindirubin-3′-oxime (BIO), a specific inhibitor of glycogen synthesis kinase-3 (GSK-3), activating the Wnt/*β*-catenin signalling pathway (Sato *et al*, 2004), was purchased from Calbiochem (San Diego, CA, USA) and was dissolved in dimethyl sulphoxide (DMSO) (Wako Pure Chemical Industries, Osaka, Japan). We used the following antibodies for immunohistochemistry and western blot analysis: monoclonal mouse anti-human Ep-CAM antibody (Abcam, Cambridge, UK), polyclonal rabbit anti-human c-MYC antibody (Cell Signaling Technology, Beverly, MA, USA) and polyclonal rabbit anti-human *β*-actin (Sigma, St Louis, MO, USA).

### Patients and specimens

In total, 30 patients with multiple liver tumours spreading to both lobes with tumour thrombi in the major branches of the portal vein, underwent palliative reduction surgery at the Osaka University Hospital as described in our earlier report (Nagano *et al*, 2007a). All 30 patients had visible tumours in the remnant liver, and received combination chemotherapy with 5-FU and IFN-*α* as described earlier (Sakon *et al*, 2002; Ota *et al*, 2005). The chemotherapeutic response was evaluated clinically according to the criteria of the Eastern Cooperative Oncology Group (Oken *et al*, 1982). In this study, responders were defined as patients with complete response or partial response; non-responders were defined as patients with stable disease or progressive disease. All aspects of our study protocol were approved by the Human Ethics Committee of Graduate School of Medicine, Osaka University, Japan. Surgical specimens were obtained from these patients. Appropriate informed consent was obtained from all patients.

For microarray analysis, we used samples of 18 cases that were positive for IFNAR2 expression, whereas no samples were available from 2 cases with insufficient quality of RNA. For reference in microarray experiment, we obtained a mixture of RNA from normal parts of the liver specimens of seven patients with liver metastases from intestinal carcinomas who were free of HBV and HCV infections. All tissues were snap-frozen into liquid nitrogen and stored at −80°C. Other samples were fixed in 10% buffered formalin, embedded in paraffin and stained with haematoxylin–eosin to study the pathological features of HCC in accordance with the classification proposed by the Liver Cancer Study Group of Japan.

### Microarray experiments

The microarray experiments were conducted according to the method described earlier (Kittaka *et al*, 2008). In brief, total RNA was purified by TRIzol agent (Invitrogen, San Diego, CA, USA), according to the instructions provided by the manufacturer. The integrity of RNA was assessed by Agilent 2100 Bioanalyzer and RNA 6000 LabChip kits (Yokokawa Analytical Systems, Tokyo, Japan). Only high-quality RNA was used for analysis. For control reference, RNAs from normal liver tissues were mixed. The reference and sample were mixed and hybridised on a microarray covering 30 336 human probes (AceGene Human 30K; DNA Chip Research Inc. and Hitachi Software Engineering Co., Kanagawa, Japan). The ratio of expression level of each gene was converted to a logarithmic scale (base 2) and the data matrix was normalised. Genes with >10% missing data values in all samples were excluded from analysis; a total of 14 473 genes out of 30 336 were available for analysis.

To detect the significant genes for resistance, we used permutation testing (Kurokawa *et al*, 2004b). The original score of each gene (signal-to-noise ratio (S2N), Si=(*μ*A−*μ*B)/(*σ*A+*σ*B), where *μ* and *σ* represent the mean and standard deviation of expression for each class, was calculated without permuting labels (responder or non-responder). The labels were randomly swapped and the values of S2N were calculated between two groups. Repetition of this permutation 10 000 times provided data matrix that was nearly the same as normal distribution. For each gene, the *P*-value was calculated from the original S2N ratio with reference to the distribution of permuted data matrix. We determined the optimal *P*-value and the informative gene set.

### Pathway analysis

We further analysed the significant genes for resistance by the Ingenuity Pathways Analysis (Ingenuity Systems, Mountain View, CA, USA; http://www.ingenuity.com). The Ingenuity Pathway Knowledge Base (IPKB) is a database of earlier published findings on mammalian biology. Canonical pathways analysis identifies the pathways that were statistically significant from the submitted data matrix from the canonical pathways of IPKB. The *P*-value of each canonical pathway is calculated using Fischer's exact test determining the probability that the association between the genes in the data set and the canonical pathway is because of chance alone.

Network analysis was conducted as described earlier (Calvano *et al*, 2005). In brief, the submitted genes that were mapped to the corresponding gene objects in the IPKB were called ‘focus genes’. The focus genes were used to generate biological networks. The Ingenuity software queries the IPKB for interactions between focus genes and then generates a set of networks. The *P*-value of each network is calculated according to the fit of the user's set of significant genes. The score of a network is displayed as a negative log of the *P*-value, indicating the probability that a collection of genes equal to or greater than the number in a network could be achieved by chance alone.

### RT–PCR analysis

Complementary DNA was generated from 1 *μ*g RNA with avian myeloblastosis virus reverse transcriptase (Promega, Madison, WI, USA) as described earlier (Damdinsuren *et al*, 2006). Quantitative real-time PCR (qRT–PCR) assays were carried out using the Light Cycler (Roche Diagnostics, Mannheim, Germany), as described earlier (Ogawa *et al*, 2004). Gene expression was measured in duplicate. The conditions set for qRT–PCR for TACSTD1, TCF3, AXIN2, MYC, CCND1 and *β*-actin were one cycle of denaturing at 95°C for 10 min, followed by 40 cycles of 95°C for 15 s, 60°C for 15 s and 72°C for 35 s, and final extension at 72°C for 10 min (or annealing at 58°C for *β*-actin). The primer sequences were as follows: TACSTD1 forward primer, 5′-TCCAGAAAGAAGAGAATGGCA-3′; TACSTD1 reverse primer, 5′-AAAGATGTCTTCGTCCCACG-3′; TCF3 forward primer, 5′-ATCTGTGTCCCATGTCCCAG-3′; TCF3 reverse primer, 5′-CCAGGGTAGGAGACTTGCAG-3′; AXIN2 forward primer, 5′-GGTGTTTGAGGAGATCTGGG-3′; AXIN2 reverse primer, 5′-TGCTCACAGCCAAGACAGTT-3′; MYC forward primer, 5′-AAGAGGACTTGTTGCGGAAA-3′; MYC reverse primer, 5′-CTCAGCCAAGGTTGTGAGGT-3′; CCND1 forward primer, 5′-AAGGCCTGAACCTGAGGAG-3′; CCND1 reverse primer, 5′-CTTGACTCCAGCAGGGCTT-3′; *β*-actin forward primer, 5′-GAAAATCTGGCACCACACCTT-3′; and *β*-actin reverse primer, 5′-GTTGAAGGTAGTTTCGTGGAT-3′.

### Immunohistochemical staining

For immunohistochemical staining of Ep-CAM expression, we used the method described earlier (Kondo *et al*, 1999) with minor modifications. Briefly, formalin-fixed, paraffin-embedded 4-*μ*m thick sections were deparaffinised, then treated with an antigen retrieval procedure and incubated in methanol containing 0.3% hydrogen peroxide to block endogenous peroxidase. The sections were incubated with normal protein block serum solution, and the biotin-blocking solution (Wako) was used as recommended by the manufacturer. Then, the sections were incubated overnight at 4°C with anti-Ep-CAM antibody as the primary antibody. After washing in phosphate-buffered saline (PBS), the sections were incubated with a biotin-conjugated secondary antibody (horse anti-mouse antibody for Ep-CAM) and with peroxidase-conjugated streptavidin. The peroxidase reaction was then developed with 0.02% 3, 30-diaminobenzidine tetrachloride (Wako) solution with 0.03% hydrogen peroxide. Finally, the sections were counterstained with Meyer's haematoxylin. For negative controls, sections were treated the same way except that they were incubated with Tris-buffered saline instead of the primary antibody.

Ep-CAM expression was assessed by two investigators (TN and NM) independently without knowledge of the corresponding clinicopathological data. Antigen expression was defined as the presence of specific staining on the surface membrane of tumour cells. Ep-CAM expression was evaluated by calculating the total immunostaining score, representing the product of the proportion score and the intensity score, as described earlier (Gastl *et al*, 2000). In brief, the proportion score described the estimated fraction of positively stained tumour cells (0, none; 1, <10%; 2, 10–50%; 3, 50–80% and 4, ⩾80%). The intensity of Ep-CAM expression was compared with staining of normal bile duct epithelium present in each section of positive control. The intensity score represented the estimated staining intensity (0, no staining; 1, weak; 2, moderate and 3, strong). The total score ranged from 0 to 12. Ep-CAM-positive cases represented those with a total score >4.

### Western blot analysis

The cells were washed with PBS and collected with a rubber scraper. After centrifugation, the cell pellets were resuspended in RIPA buffer (25 mM Tris (pH 7.5), 50 mM NaCl, 0.5% sodium deoxycholate, 2% Nonidet P-40, 0.2% sodium dodecyl sulphate, 1 mM phenylmethylsulphonyl fluoride and 500 KIE ml^−1^ ‘Trasylol’ proteinase inhibitor (Bayer LeverKusen, Germany)) with phosphatase inhibitor (Sigma). The extracts were centrifuged and the supernatant fraction was collected. Western blot analysis was carried out as described earlier (Kondo *et al*, 2005).

### Luciferase reporter assay

The reporter assay kit was purchased from SA Biosciences (Frederick, MD, USA) to evaluate TCF/LEF transcriptional activity and is used according to the instructions provided by the manufacturer. In brief, 2 × 10^4^ cells per well were added in triplicate to a 96-well microplate, and 24 h later, cells were transiently transfected with the transcription factor-responsive reporter or negative control by the Lipofectamine 2000 reagent (Invitrogen). Culture media were changed 16 h after transfection, and the transfected cells were treated with various concentrations of BIO (0–5 nM). After 24 h treatment, luciferase activity was measured with the Dual-Luciferase Assay System (Promega) using microplate luminometer, microlumat LB96P (Berthold Technologies, Calmbacher, Germany). The Firefly luciferase activity, indicating TCF-dependent transcription, was normalised to the *Renilla* luciferase activity as an internal control to obtain the relative luciferase activity.

### Growth-inhibitory assays with 5-FU and IFN-*α*

The growth inhibitory assay was assessed by the 3-(4-,5-dimethylthiazol-2-yl)-2,5-diphenyl tetrazolium bromide (MTT) (Sigma) assay as described earlier (Eguchi *et al*, 2000). The tested concentrations of 5-FU were 0.05, 0.5 and 5 *μ*g ml^−1^, and those of IFN-*α* were 50, 500 and 5000 U ml^−1^. The cells were incubated in a medium containing variable concentrations of 5-FU and IFN-*α* with DMSO or 5 nM BIO for 48 h. The proportion of cells incubated without drugs was defined as 100% viability.

### DNA synthesis-inhibition assay

DNA synthesis inhibition was assessed by bromodeoxyuridine (BrdU) incorporation rate using the Cell Proliferation enzyme-linked immunosorbent assay (ELISA)-Chemiluminescent kit (Roche Applied Science, Indianapolis, IN, USA) according to the protocol provided by the manufacturer. In brief, HuH7 cells (1 × 10^4^ per well) were seeded in triplicate into 96-well microplate. After treatment with control, 5-FU alone (5 *μ*g ml^−1^), IFN-*α* alone (5000 U ml^−1^) and combination of 5-FU and IFN-*α*, with or without BIO (5 nM), the plates were incubated at 37°C under 5% CO_2_ for 24 h. Then cells were labelled for 2 h with BrdU. Chemiluminescent signals were detected on the microplate luminometer (microlumat LB96P, Berthold Technologies).

### Cell cycle analysis

Flow cytometric analysis was carried out as described earlier (Eguchi *et al*, 2000). In brief, cells were washed with PBS and then fixed in 70% cold ethanol. Propidium iodide (Sigma) and RNase (Sigma) were added for 30 min at 37°C. Samples were filtered, and data were acquired with a FACSort (Becton Dickinson Immunocytometry Systems, San Jose, CA, USA). Analysis of the cell cycle was carried out using ModFIT software (Becton Dickinson Immunocytometry Systems).

### Statistical analysis

Clinicopathological indicators were compared using *χ*^2^-test, whereas continuous variables were compared using the Student's *t*-test. Survival curves were computed using the Kaplan–Meier method, and differences between survival curves were compared using the log-rank test. To evaluate the risk associated with the prognostic variables, the Cox model with determination of the hazard ratio was applied; a 95% confidence interval was adopted. All statistical analyses were calculated using the SPSS software (version 11.0.1 J, SPSS Inc., Chicago, IL, USA), and *P*-value <0.05 was considered statistically significant.

## Results

### Patients’ characteristics

The characteristics of the 30 HCC patients are shown in Table 1. A total of 10 patients were IFNAR2-negative and 20 patients were IFNAR2-positive. In 20 cases with positive IFNAR2, 10 patients were classified as responders; the remaining 10 patients were classified as non-responders. All patients with negative IFNAR2 were non-responders. We have earlier reported that IFNAR2 expression correlated significantly with the response to IFN-*α*/5-FU therapy (Ota *et al*, 2005; Nagano *et al*, 2007a). A larger proportion of responders were infected with HCV than non-responders. But all other analysed parameters were comparable among these groups and there were no significant differences in these parameters.

### Microarray analysis and pathway analysis

Genes with significant *P*-values (*P*<0.001) were defined by the random permutation test. These differentially expressed 161 genes were selected as informative gene set and are listed in Table 2. The status of gene expression was defined as expression in non-responders compared with responders. Of the total, 98 genes were relatively upregulated in the responder group and 63 genes were relatively downregulated.

**Figure 1:**
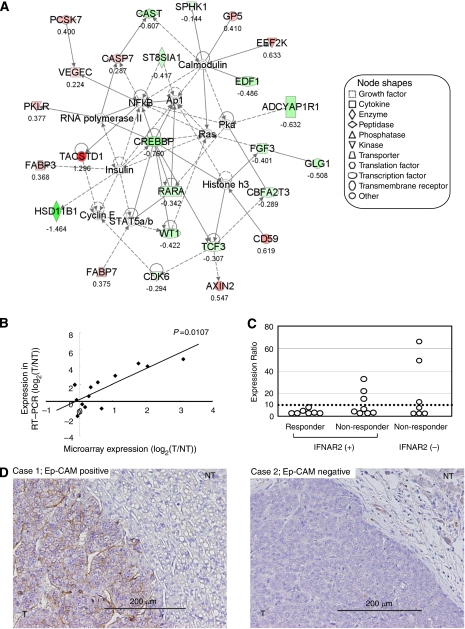
(**A**) Gene network of genes related to resistance to IFN-*α*/5-FU combination therapy. This network with the highest score consisted of 35 molecules in 19 focus molecules (red or green colour) and 16 interconnecting molecules (not coloured). The network included AXIN2, TCF3, RARA, CREBBP and TACSTD1, which are all associated with Wnt/*β*-catenin signalling. Each value of gene expression correlated directly with the intensity of the node colour. Red: upregulation in non-responders, green: downregulation in non-responders. The ratio of expression of each gene (non-responders/responders) is indicated below each node. (**B**) The expression levels determined by quantitative RT–PCR analysis correlated significantly with the microarray data. The Pearson correlation coefficient (*P*-value) for TACSTD1 were 0.668 (*P*=0.0107) (**C**) Among non-responders with IFNAR2-positive HCC or IFNAR2-negative HCC, the TACSTD1 expression ratio was higher in several cases than that in responders with IFNAR2-positive HCC. (**D**) Immunohistochemical staining for Ep-CAM in representative cases. Left panel: the majority of tumour cells showing staining for Ep-CAM on the plasma membrane. Right panel: tumour cells were negative for Ep-CAM. T, tumour lesion; NT, non-tumour lesion. (Magnification, × 200). The colour reproduction of this figure is available on the html full text version of the paper.

Then we carried out the canonical pathway analysis of the 161 genes using the software Ingenuity. Eight canonical pathways were identified as pathways that significantly influenced the resistance of IFN-*α*/5-FU combination therapy in 161 informative genes (Table 3). We also simultaneously carried out network analysis of the same informative genes set. A total of 14 networks were identified, and these networks were ranked by the score on a *P*-value calculation, which ranged from 2 to 55. Then, we selected one network with the highest score. The network with the highest score consisted of 35 molecules in 25 focus molecules and 11 interconnecting molecules (Figure 1A). This network included AXIN2, TCF3, RARA, CREBBP and TACSTD1, which were all associated with Wnt/*β*-catenin signalling identified by the canonical pathway analysis. In recent reports, Wnt/*β*-catenin signalling has been shown to mediate radiation resistance and chemotherapy resistance of various malignancies. In the Wnt/*β*-catenin signalling-related genes, TACSTD1 was most highly upregulated in the non-responders at the level of transcription.

### TACSTD1 expression by RT–PCR and correlation with microarray data

Next, we examined the correlation between the expression data of gene expression and qRT–PCR of TACSTD1 to verify the microarray expression data. qRT–PCR analysis was carried out on 13 HCC tissue samples with positive expression of IFNAR2. Individual mRNA levels were normalised to *β*-actin and expressed relative to those in a mixture of seven normal livers. In the 13 IFNAR2-positive samples, TACSTD1 expression correlated significantly with the microarray data (Figure 1B). The Pearson correlation coefficient (*P*-value) for TACSTD1 was 0.668 (*P*=0.0107). We then analysed TACSTD1 expression according to the clinical response to IFN-*α*/5-FU combination therapy. TACSTD1 expression was higher in several non-responders with IFNAR2-positive HCC or IFNAR2-negative HCC, compared with responders with IFNAR2-positive HCC (Figure 1C). Using a cut-off value of 10 for TACSTD1 expression ratio, it was possible to exclude some non-responders from patients with IFNAR2-positive HCC.

### Immunohistochemical staining for Ep-CAM

We examined the Ep-CAM expression in 30 HCC patients who underwent palliative reduction surgery. In tumour lesions, Ep-CAM staining was specifically observed on the plasma membrane of cancer cells. In Figure 1D (left), strong Ep-CAM expression was noted in 80% of cancerous tissue in the representative case of non-responders with IFNAR2-negative HCC. On the other hand, no Ep-CAM expression was evident in the representative case of IFNAR2-positive responders (Figure 1D, right). Among the 30 patients examined, Ep-CAM expression was observed in six (20%). It is important that Ep-CAM expression was associated with resistance to IFN-*α*/5-FU therapy, and no Ep-CAM expression was noted in the responders (Table 4). However, the difference in the expression rate was not significant probably because of the small sample size (*P*=0.0528). In non-tumour lesions, Ep-CAM staining was observed in a few scattered cells and proliferating bile duct epithelium showed positive expression.

Analysis of the degree of Ep-CAM expression in tumour lesions showed five (16.7%) samples negative for Ep-CAM expression (score 0), 19 (63.3%) with weak expression (score 1–4), four (13.3%) stained moderately (score 6–8) and two (6.7%) samples exhibited strong Ep-CAM expression (score 9–12). These results suggest that Ep-CAM expression in advanced HCC could be a potentially useful marker for resistance to IFN-*α*/5-FU combination therapy.

### Ep-CAM expression and activation of Wnt/*β*-catenin signalling by BIO

**Figure 2:**
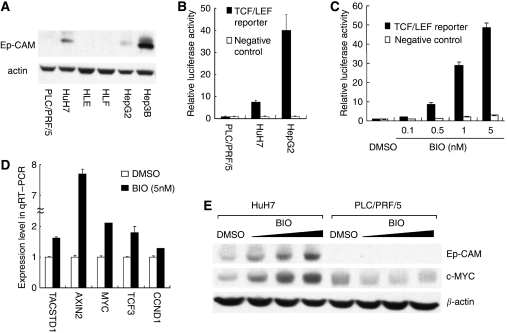
Changes in Ep-CAM expression and TCF/LEF transcription activity after treatment with BIO. (**A**) Western blot analysis of Ep-CAM in human hepatoma cell lines. Expression of Ep-CAM was positive in HuH7, HepG2 and Hep3B cell lines, but not in PLC/PRF/5, HLE and HLF. (**B**) Luciferase reporter assay of PLC/PRF/5, HuH7 and HepG2 cells. Relative luciferase activities were high in both Ep-CAM-positive HuH7 cells and HepG2 cells, whereas very low in Ep-CAM-negative PLC/PRF/5 cells. The assay was conducted in triplicate and results are shown as mean±s.d. (**C**) Luciferase reporter assay of HuH7 cells treated with various concentrations of BIO for 24 h. Treatment with 5 nM induced 48.6-fold increase in relative luciferase activity compared with DMSO. The assay was conducted in triplicate and results are shown as mean±s.d. (**D**) qRT–PCR analysis of HuH7 cells treated with BIO for 24 h. BIO increased the expression levels of TACSTD1, AXIN2, MYC, TCF3 and CCND1 compared with DMSO. Data are mean±s.d. values of gene expression measured in duplicate. (**E**) Western blot analysis of HCC cell lines treated with BIO for 48 h. The expression of Ep-CAM and c-MYC increased in a BIO dose-dependent manner in HuH7 cells, but not in PLC/PRF/5 cells.

We analysed the protein expression level of Ep-CAM in hepatoma cell lines. Western blot analysis using an anti-Ep-CAM antibody confirmed the positive expression of Ep-CAM in three of the six cell lines (HuH7, HepG2 and Hep3B), whereas PLC/PRF/5, HLE and HLF were negative (Figure 2A). We earlier reported strong IFNAR2 expression in PLC/PRF/5 cells and weak IFNAR2 expression in HuH7 cells (Eguchi *et al*, 2000). We transfected a TCF/LEF reporter into PLC/PRF/5, HuH7 and HepG2 cells to evaluate TCF/LEF transcriptional activity, representing the activity of Wnt/*β*-catenin signalling pathway. We found that the luciferase activities were high in both Ep-CAM-positive HuH7 cells and HepG2 cells, whereas very low in Ep-CAM-negative PLC/PRF/5 cells (Figure 2B). And, HepG2 cell line was reported to have mutation and activated *β*-catenin (de La Coste *et al*, 1998). For these reasons, we used the cell line HuH7 to investigate how Wnt/*β*-catenin signalling affected on the growth-inhibitory effect of IFN-*α*/5-FU. In the next step, we examined whether Wnt/*β*-catenin signalling can be activated in HuH7 cells when treated with various concentrations of specific GSK-3 inhibitor, BIO. HuH7 cells treated with BIO showed a substantial, dose-dependent increase in TCF/LEF reporter activity. Consequently, treatment with BIO at 0.5, 1 and 5 nM induced 8.6-, 29.1-, and 48.6-fold increases in relative luciferase activity compared with HuH7 cells treated by DMSO, respectively (Figure 2C). To examine the effects of BIO on the expression of Wnt/*β*-catenin signalling targeted genes, qRT–PCR analysis of five targeted genes (TACSTD1, AXIN2, MYC, TCF3 and CCND1) was carried out in HuH7 cells after 24 h treatment with BIO (5 nM). The concentration of BIO was selected on the basis of the results of luciferase reporter assay. Treatment with BIO increased the mRNA expression of targeted genes from 1.3-fold to 7.6-fold compared with cells treated with DMSO (Figure 2D). In western blot analysis, the expression levels of Ep-CAM and c-MYC increased in a BIO dose-dependent manner in HuH7 cells, but not in PLC/PRF/5 cells (Figure 2E).

### Growth inhibition assay and reduction of growth-inhibitory effect of 5-FU and/or IFN-*α* treatment

**Figure 3:**
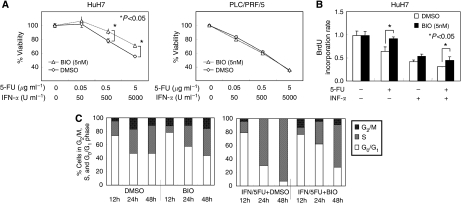
(**A**) Changes in susceptibility to the combination of 5-FU and IFN-*α* was measured by MTT assay. All cells were incubated with various concentrations of 5-FU and IFN-*α* and with BIO (5 nM) or DMSO. When BIO was combined with IFN-*α*/5-FU, it significantly reduced the growth inhibitory effects of IFN-*α*/5-FU in HuH7 cells, but not PLC/PRF/5 cells. The viability of cells incubated without drugs was defined as 100% and data are shown as mean±s.d. (**B**) DNA synthesis-inhibition assay of HuH7 cell was assessed by BrdU incorporation rate. Cells were incubated with 5-FU and/or IFN-*α* and with BIO (5 nM) or DMSO. In both cell lines, the addition of BIO with 5-FU alone and IFN-*α*/5-FU significantly reduced the inhibitory effects of IFN-*α*/5-FU on DNA synthesis. Data was measured in triplicate and are shown as mean±s.d. (**C**) Results of flow cytometric analysis of HuH7 cells treated with BIO and/or IFN-*α*/5-FU combination. Data represent percentages of cells in G_2_/M, S and G_0_/G_1_ phases of the cell cycle. Concurrent use of BIO with IFN-*α*/5-FU delayed the accumulation of S-phase fraction.

Next, we investigated the role of activation of Wnt/*β*-catenin signalling in the reduction of the growth-inhibitory effect of IFN-*α*/5-FU. The growth of HCC cells (PLC/PRF/5 and HuH7 cell lines) was suppressed by 5-FU and IFN-*α* in a dose-dependent manner. Concurrent addition of BIO and IFN-*α*/5-FU to the cell cultures significantly reduced the growth-inhibitory effects of IFN-*α*/5-FU in HuH7 cells. In HuH7 cells, the growth inhibitory effects of IFN-*α*/5-FU without BIO were 22.3±2.8% at 0.5 *μ*g ml^−1^ 5-FU and 500 U ml^−1^ IFN-*α*, and 44.6±0.9% at 5 *μ*g ml^−1^ and 5000 U ml^−1^. Concurrent addition of BIO decreased the growth inhibitory effect to 8.6±3.9% (*P*=0.0012; 0.5 *μ*g ml^−1^ of 5-FU and 500 U ml^−1^ for IFN-*α*) and 29.0±2.0% (*P*<0.0001, 5 *μ*g ml^−1^ for 5-FU and 5000 U ml^−1^ for IFN-*α*) of control cells. In contrast, no change in the growth-inhibitory effect was found in PLC/PRF/5 cell line (Figure 3A). We also investigated the effects of BIO when combined with 5-FU alone or IFN-*α* alone in HuH7 cells. The combination of BIO and 5-FU alone and BIO and IFN-*α* exhibited reduced anti-proliferative effects (data not shown).

### Activation of Wnt/*β*-catenin signalling interferes with the inhibitory effect of IFN-*α*/5-FU on DNA synthesis

To investigate whether activation of Wnt/*β*-catenin signalling is involved in the reduction of the growth inhibitory effects of IFN-*α*/5-FU, we evaluated the effects of BIO and IFN-*α*/5-FU on DNA synthesis using a BrdU-based cell proliferation ELISA. In HuH7 cells, the BrdU incorporation rates (representing DNA synthesis) in cultures treated with 5-FU alone and IFN-*α*/5-FU were 0.649±0.052 and 0.312±0.004, respectively. Activation of Wnt/*β*-catenin signalling by BIO resulted in a significant interference with the inhibitory effect of IFN-*α*/5-FU on DNA synthesis; the BrdU incorporation rates in cells cultured with BIO and 5-FU alone and with BIO and IFN-*α*/5-FU were significantly increased to 0.928±0.020 (*P*=0.002) and 0.458±0.037 (*P*=0.007) (Figure 3B).

### Cell cycle assay

Finally, we used flow cytometric analysis to examine changes in cell cycle progression in cultures treated with BIO and IFN-*α*/5-FU. In cultures refed with serum plus 5-FU and IFN-*α*, the distribution of cells at different cell cycles was similar to that of cells treated with DMSO at 12 h. Thereafter, HuH7 cell lines treated with 5-FU and IFN-*α* showed accumulation of cells in S-phase and a gradual increase in S-phase fraction from 24 to 48 h. Addition of BIO and IFN-*α*/5-FU to the cell cultures delayed the accumulation of S-phase fraction. Marked accumulation of cells in S-phase (24 h; 69.4% and 48 h; 92.9%) was noted in cultures of cells treated with IFN-*α*/5-FU, whereas the percentage of cells in S-phase in cultures of BIO and IFN-*α*/5-FU decreased to 34.9% and 62.9% at the respective time points (Figure 3C).

## Discussion

Gene expression profiling analyses represent a high-throughput approach to dissect the biology underlining resistance to anticancer drugs in malignancies. We earlier identified a 63-gene set that could predict the response to IFN-*α*/5-FU combination therapy using a small-scale PCR array system of a total of 2666 genes (Kurokawa *et al*, 2004a). In this study, we used advanced technology with human whole genes analysis covering 30,336 human probes compared with the PCR array system. This comprehensive analysis allowed us to identify the biological actions of IFN-*α*/5-FU combination therapy. Furthermore, creating biological networks from comprehensive gene expression profiling could be useful for discovering certain targeted molecules and pathways. In fact, we reported recently genome-wide expression profiling of 100 HCC tissues using this network analysis, Ingenuity Pathway Analysis and identified novel targeted molecules related to specific signalling pathways (Kittaka *et al*, 2008).

In this study, gene expression profiling and pathway analysis identified Wnt/*β*-catenin signalling as a significant canonical pathway. The Wnt/*β*-catenin-signalling pathway plays an important role in the development of various malignancies, as well as cell proliferation and differentiation in several adult stem cells (Barker and Clevers, 2006; Klaus and Birchmeier, 2008). It has been also shown that anti-cancer drugs or irradiation often kill tumour cells, yet putative cancer stem/progenitor cells are resistant to these agents (Jamieson *et al*, 2004; Woodward *et al*, 2007; Klaus and Birchmeier, 2008). Cancer stem/progenitor cells provide an attractive explanation for chemotherapy-induced tumour remission as well as relapse. Analysis of the molecular and signalling mechanism of resistance of cancer stem/progenitor cells should be important for the development of new therapeutic strategies. Recent studies showed that the Wnt/*β*-catenin pathway plays a role in radiation and/or chemotherapy resistance of various malignancies such as leukaemia, head and neck tumours, prostate cancer and HCC (Jamieson *et al*, 2004; Ohigashi *et al*, 2005; Chang *et al*, 2008; Yang *et al*, 2008). In this study, we also showed that activation of Wnt/*β*-catenin signalling by a specific GSK-3 inhibitor in hepatoma cell lines decreased the susceptibility to IFN-*α*/5-FU through a reduction in their DNA synthesis inhibitory effects and regulation of cell cycle progression. We have already reported the mechanisms of the anti-proliferative effects of IFN-*α*/5-FU combination therapy, including regulation of cell cycle progression by increasing S-phase fraction (Eguchi *et al*, 2000), induction of apoptosis through IFNAR2, by downregulating Bcl-xl and by Fas/FasL pathway (Kondo *et al*, 2005; Damdinsuren *et al*, 2007; Nakamura *et al*, 2007; Nagano *et al*, 2007a), modulation of the immune response by inducing the TRAIL/TRAIL-receptor pathway (Yamamoto *et al*, 2004) and inhibition of tumour angiogenesis (Wada *et al*, 2007). In addition to the above mechanisms related to their anti-proliferative effects, this study showed that activation of Wnt/*β*-catenin signalling resulted in reduction of the inhibitory effects of IFN-*α*/5-FU on DNA synthesis, by decreasing the accumulation of cells in S-phase. With regard to the apoptotic effect of the combination therapy, it is reported that Wnt/*β*-catenin signalling is closely linked to JAK–STAT signalling (Yamashina *et al*, 2006), and regulates STAT3 expression, thus enhancing cell growth and anti-apoptotic activity of various cancer cells (Kusaba *et al*, 2007). We earlier reported that IFN-*α*/5-FU combination therapy increased the frequency of apoptosis in PLC/PRF/5 cells, but only minimally in HuH7 cells (<1%) (Eguchi *et al*, 2000). We also analysed the influence of activation of Wnt/*β*-catenin signalling on the apoptotic effects of IFN-*α*/5-FU combination therapy, but no significant change was observed in HuH7 cells probably because of the low rate of apoptosis. Further studies are needed to examine the molecular mechanisms of Wnt/*β*-catenin signalling-related enhancement of resistance to IFN-*α*/5-FU combination therapy.

Activation of the Wnt/*β*-catenin signalling pathway is reported in various diseases including many malignancies (Moon *et al*, 2004; Reya and Clevers, 2005; Branda and Wands, 2006). The ideal method for detecting the signalling activity in human tissues remains controversial (Giles *et al*, 1980). A recent study identified Ep-CAM as a novel Wnt/*β*-catenin signalling target gene in HCC cell lines, which could also serve as a biomarker (Yamashita *et al*, 2007). Ep-CAM is a first tumour-associated antigen and encoded by the TACSTD1 gene (Herlyn *et al*, 1979; Litvinov *et al*, 1994). In liver neoplasia, Ep-CAM is expressed in almost all cholangiocarcinomas, whereas 14% of HCCs manifested the expression, which seems to be more pronounced in poorly differentiated HCCs (Breuhahn *et al*, 2006). Ep-CAM-positive HCC displayed a molecular signature with features of hepatic progenitor cells, including the presence of known stem/progenitor markers such as c-kit, cytokeratin 19. In earlier studies, we showed that the expression of IFNAR2 is the only significant predictor of clinical outcome of IFN-*α*/5-FU combination therapy (Ota *et al*, 2005; Nagano *et al*, 2007a). On the basis of the present results on 30 HCC tissue samples, Ep-CAM seems to be another predictor of IFN-*α*/5-FU combination therapy. Further studies are needed to validate this result using larger sample numbers to establish the precise clinical response to IFN-*α*/5-FU combination therapy.

In summary, we showed that activation of Wnt/*β*-catenin signalling enhanced the resistance to IFN-*α*/5-FU therapy by reducing the inhibitory effects of these drugs on DNA synthesis and regulation of cell cycle progression *in vitro*. Furthermore, the results identified Ep-CAM expression in HCC tissue specimen as a potential biological marker for resistance to IFN-*α*/5-FU combination therapy.

## Figures and Tables

**Table 1:** Clinicopathological characteristics of responders and non-responders

	**IFNAR2-positive**		**IFNAR2-negative**	
	**Responders (*n*=10)**	**Non-responders (*n*=10)**	**Non-responders (*n*=10)**	***P*-value**
*Age (year)*				NS
<60	6	7	5	
⩾60	4	3	5	
				
*Sex*				NS
Male	9	9	9	
Female	1	1	1	
				
*HBV infection*				NS
Present	6	8	7	
Absent	4	2	3	
				
*HCV infection*				0.0180
Present	7	1	3	
Absent	3	9	7	
				
*Child-pugh score*				NS
A	7	7	5	
B, C	3	3	5	
				
*Liver cirrhosis*				NS
Present	4	7	3	
Absent	6	3	7	
				
*α*-fetoprotein (ng ml^−1^)				NS
<300	5	1	3	
⩾300	5	9	7	
				
*Tumour size (cm)*				NS
<5	2	3	2	
⩾5	8	7	8	
				
*Histological grade*				NS
Moderately differentiated	1	0	0	
Poorly differentiated	9	8	9	
Undifferentiated	0	2	1	
				
*IFNAR2 expression*				<0.0001
0	0	0	10	
1	8	10	0	
2	2	0	0	

HBV=hepatitis B virus; HCV=hepatitis C virus; IFNAR2=type I interferon receptor 2.

**Table 2:** List of informative 161 genes defining responders and non-responders

**Rank**	**Status**	**Gene symbol**	**Gene name**	**Ref Seq ID**
1	Down	—	dj329l24.3 (member of mcm2/3/5 family)	—
2	Down	CREBBP	CREB-binding protein (Rubinstein-Taybi syndrome)	NM_004380
3	Down	C20orf116	Chromosome 20 open reading frame 116	NM_023935
4	Down	—	Ensembl genscan prediction	—
5	Down	SLC25A40	Solute carrier family 25, member 40	NM_018843
6	Up	ZNF598	Zinc-finger protein 598	NM_178167
7	Down	RCOR1	REST corepressor 1	NM_015156
8	Up	TACSTD1	Tumour-associated calcium signal transducer 1	NM_002354
9	Down	ZNF397	Zinc-finger protein 397	NM_032347
10	Down	RTP3	Receptor (chemosensory) transporter protein 3	NM_031440
11	Up	AARS2	Alanyl-tRNA synthetase 2, mitochondrial (putative)	NM_020745
12	Down	RARA	Retinoic acid receptor, alpha	NM_000964
13	Up	—	DNA segment on chromosome 4 (unique) 234 expressed sequence	NM_014392
14	Up	CASP7	Caspase 7, apoptosis-related cysteine peptidase	NM_033339
15	Up	AZIN1	Antizyme inhibitor 1	NM_148174
16	Down	—	Ensembl prediction	—
17	Down	—	Ensembl genscan prediction	—
18	Up	PABPN1	Poly(A)-binding protein, nuclear 1	—
19	Down	NDUFS7	NADH dehydrogenase (ubiquinone) Fe-S protein 7, 20 kDa (NADH-coenzyme Q reductase)	NM_024407
20	Down	C9orf142	Chromosome 9 open reading frame 142	NM_183241
21	Down	—	Ensembl genscan prediction	—
22	Up	SNX21	Sorting nexin family member 21	NM_001042632
23	Down	C12orf47	Chromosome 12 open reading frame 47	XR_017874
24	Down	CCRL1	Chemokine (C–C motif) receptor-like 1	NM_178445
25	Down	GLG1	Golgi apparatus protein 1	NM_012201
26	Down	MRPS21	Mitochondrial ribosomal protein S21	NM_018997
27	Up	ABCA2	ATP-binding cassette, sub-family A (ABC1), member 2	NM_001606
28	Up	AXIN2	Axin 2 (conductin, axil)	NM_004655
29	Down	—	Ensembl genscan prediction	—
30	Down	NOXA1	NADPH oxidase activator 1	NM_006647
31	Down	COX4I1	Cytochrome c oxidase subunit IV isoform 1	NM_001861
32	Up	PCSK7	Proprotein convertase subtilisin/kexin type 7	XM_001128785
33	Up	FABP3	Fatty acid-binding protein 3, muscle and heart (mammary-derived growth inhibitor)	NM_004102
34	Down	C20orf111	Chromosome 20 open reading frame 111	NM_016470
35	Down	CAST	Calpastatin	NM_173061
36	Down	C12orf47	Chromosome 12 open reading frame 47	XR_017874
37	Up	—	Hypothetical protein xp_032244	—
38	Down	—	Ensembl genscan prediction	—
39	Up	PCDHA1	Protocadherin alpha 1	NM_018900
40	Down	MATK	Megakaryocyte-associated tyrosine kinase	NM_002378
41	Up	—	Hypothetical protein xp_051475	—
42	Up	UBE2Q1	Ubiquitin-conjugating enzyme E2Q (putative) 1	NM_017582
43	Up	GPATCH4	G patch domain containing 4	NM_182679
44	Down	PARP2	Poly (ADP-ribose) polymerase family, member 2	NM_005484
45	Down	HAL	Histidine ammonia-lyase	NM_002108
46	Up	ASCC3	Activating signal cointegrator 1 complex subunit 3	NM_006828
47	Down	KRTAP9-8	Keratin-associated protein 9–8	NM_031963
48	Up	MAGED4B	Melanoma antigen family D, 4B	NM_030801
49	Down	—	Hypothetical LOC339123	NM_001005920
50	Down	SPHK1	Sphingosine kinase 1	NM_021972
51	Up	—	Partial ighv ig h-chain v-region clone a81	—
52	Up	CCDC109A	Coiled-coil domain containing 109A	NM_138357
53	Up	GPR139	G protein-coupled receptor 139	NM_001002911
54	Up	C1orf78	Chromosome 1 open reading frame 78	NM_018166
55	Down	LRRC50	Leucine rich repeat containing 50	—
56	Down	FAM125B	Family with sequence similarity 125, member B	NM_033446
57	Down	IFT52	Intraflagellar transport 52 homolog (Chlamydomonas)	NM_016004
58	Down	C3orf36	Chromosome 3 open reading frame 36	NM_025041
59	Up	GUCA1B	Guanylate cyclase activator 1B (retina)	NM_002098
60	Down	EDF1	Endothelial differentiation-related factor 1	NM_003792
61	Down	CCDC69	Coiled-coil domain containing 69	NM_015621
62	Down	NDUFS6	NADH dehydrogenase (ubiquinone) Fe-S protein 6, 13 kDa (NADH-coenzyme Q reductase)	NM_004553
63	Up	CD93	CD93 molecule	NM_012072
64	Down	—	Ensembl genscan prediction	—
65	Down	ENO2	Enolase 2 (gamma, neuronal)	NM_001975
66	Down	CDCP2	CUB domain-containing protein 2	—
67	Down	—	Ensembl genscan prediction	—
68	Up	—	Similar to helicase-like protein nhl	—
69	Down	FOXN3	Forkhead box N3	NM_005197
70	Down	DEF8	Differentially expressed in FDCP 8 homolog (mouse)	NM_207514
71	Up	—	Hypothetical protein xp_034013	—
72	Down	TNS3	Tensin 3	NM_022748
73	Up	FAM40A	Family with sequence similarity 40, member A	NM_033088
74	Down	PRDM7	PR domain containing 7	NM_052996
75	Up	—	Hypothetical protein xp_039419	—
76	Up	NNMT	Nicotinamide N-methyltransferase	NM_006169
77	Up	RPL9	Ribosomal protein L9	—
78	Up	ITM2C	Integral membrane protein 2C	NM_030926
79	Up	—	Ensembl genscan prediction	—
80	Up	BEX4	BEX family member 4	XM_936467
81	Up	CSNK1G1	Casein kinase 1, gamma 1	NM_022048
82	Up	EEF2K	Eukaryotic elongation factor-2 kinase	NM_013302
83	Down	DNAJC8	DnaJ (Hsp40) homolog, subfamily C, member 8	—
84	Up	GP5	Glycoprotein V (platelet)	NM_004488
85	Down	DPH3	DPH3, KTI11 homolog (*S. cerevisiae*)	NM_001047434
86	Down	—	Ensembl genscan prediction	—
87	Up	RPS21	Ribosomal protein S21	—
88	Down	—	Kiaa1658 protein	—
89	Down	ADCYAP1R1	Adenylate cyclase activating polypeptide 1 (pituitary) receptor type I	NM_001118
90	Down	C5orf25	Chromosome 5 open reading frame 25	XR_015120
91	Down	—	PRO0132 protein	NR_002763
92	Down	FGF3	Fibroblast growth factor 3 (murine mammary tumour virus integration site (v-int-2) oncogene homolog)	NM_005247
93	Up	FABP7	Fatty acid-binding protein 7, brain	NM_001446
94	Down	HSD11B1	Hydroxysteroid (11–beta) dehydrogenase 1	NM_181755
95	Up	—	Chondroitin sulphate glucuronyltransferase	NM_019015
96	Down	OPTN	Optineurin	NM_001008213
97	Up	—	Erythroid differentiation-related factor 2	—
98	Down	—	Truncated alpha ig h-chain of disease patient har	—
99	Down	WT1	Wilms tumour 1	NM_024425
100	Down	C8G	Complement component 8, gamma polypeptide	NM_000606
101	Down	—	pro1454	—
102	Down	CADM1	Cell adhesion molecule 1	NM_001098517
103	Down	GH1	Growth hormone 1	—
104	Down	DNPEP	Aspartyl aminopeptidase	NM_012100
105	Up	—	Actin-like gene	—
106	Down	—	Ensembl genscan prediction	—
107	Down	—	Ensembl genscan prediction	—
108	Up	INTS4	Integrator complex subunit 4	NM_033547
109	Down	SRA1	Steroid receptor RNA activator 1	NM_001035235
110	Down	—	Ensembl genscan prediction	—
111	Up	RPS25	Ribosomal protein S25	NM_001028
112	Down	—	KIAA1450 protein	NM_020840
113	Up	SH2D3C	SH2 domain containing 3C	NM_170600
114	Down	NDUFA12	NADH dehydrogenase (ubiquinone) 1 alpha subcomplex, 12	NM_018838
115	Down	—	NEFA-interacting nuclear protein NIP30	NM_024946
116	Down	TDRD1	tudor domain containing 1	NM_198795
117	Down	—	14a9ct dna sequence	—
118	Up	FBXW11	F-box and WD repeat domain containing 11	NM_012300
119	Down	CBFA2T3	Core-binding factor, runt domain, alpha subunit 2; translocated to, 3	NM_005187
120	Down	TCF3	Transcription factor 3 (E2A immunoglobulin enhancer-binding factors E12/E47)	NM_003200
121	Down	LASS5	LAG1 homolog, ceramide synthase 5	NM_147190
122	Down	—	Ensembl genscan prediction	—
123	Up	ACTR1B	ARP1 actin-related protein 1 homolog B, centractin beta (yeast)	NM_005735
124	Down	—	Hypothetical protein mgc5566	—
125	Up	RPS4X	Ribosomal protein S4, X-linked	XR_019325
126	Down	CDK6	Cyclin-dependent kinase 6	NM_001259
127	Up	AVIL	Advillin	—
128	Down	—	Hypothetical protein xp_043732	—
129	Down	C1orf136	Chromosome 1 open reading frame 136	—
130	Down	—	Hypothetical protein xp_043783	—
131	Down	—	Ews-fli-1	—
132	Up	CDC42BPG	CDC42-binding protein kinase gamma (DMPK-like)	NM_017525
133	Down	—	Ensembl genscan prediction	—
134	Down	GDAP1L1	Ganglioside-induced differentiation-associated protein 1-like 1	NM_024034
135	Up	C12orf4	Chromosome 12 open reading frame 4	NM_020374
136	Up	KIAA0415	KIAA0415	NM_014855
137	Down	PDLIM2	PDZ and LIM domain 2 (mystique)	NM_198042
138	Down	KHK	Ketohexokinase (fructokinase)	NM_006488
139	Down	SLC36A1	Solute carrier family 36 (proton/amino acid symporter), member 1	NM_078483
140	Up	—	Hypothetical protein xp_050311	—
141	Up	TBRG4	Transforming growth factor *β* regulator 4	NM_199122
142	Down	—	Rearranged vk3 of Hodgkin cell line	—
143	Up	CD59	CD59 molecule, complement regulatory protein	NM_203330
144	Down	PEX26	Peroxisome biogenesis factor 26	—
145	Up	VEGFC	Vascular endothelial growth factor C	NM_005429
146	Down	DTX2	Deltex homolog 2 (*Drosophila*)	XM_941785
147	Up	ELAVL3	ELAV (embryonic lethal, abnormal vision, *Drosophila*)-like 3 (Hu antigen C)	NM_032281
148	Up	BSDC1	BSD domain containing 1	NM_018045
149	Down	FUBP3	Far upstream element (FUSE)-binding protein 3	XM_001128545
150	Down	CCDC48	Coiled-coil domain containing 48	NM_024768
151	Down	EPHA6	EPH receptor A6	NM_001080448
152	Down	ST8SIA1	ST8 alpha-N-acetyl-neuraminide alpha-2,8-sialyltransferase 1	NM_003034
153	Down	MKKS	McKusick-Kaufman syndrome	NM_018848
154	Down	MGA	MAX gene associated	NM_001080541
155	Up	—	Hypothetical protein xp_040140	—
156	Down	—	Hypothetical protein xp_043452	—
157	Up	MMP20	Matrix metallopeptidase 20 (enamelysin)	NM_004771
158	Up	SLC23A2	Solute carrier family 23 (nucleobase transporters), member 2	NM_005116
159	Up	GABARAPL1	GABA(A) receptor-associated protein like 1	NM_031412
160	Down	—	Ensembl genscan prediction	—
161	Up	PKLR	Pyruvate kinase, liver and RBC	NM_000298

Ranking was according to absolute value of signal-to-noise ratio.

Status was defined as expression in non-responders compared with responders.

**Table 3:** List of significant pathways from 161 informative genes by canonical pathway analysis

**Pathway**	***P*-value**
Ubiquinone biosynthesis	0.0004
Oxidative phosphorylation	0.0074
Mitochondrial dysfunction	0.0095
FXR/RXR activation	0.0162
Wnt/*β*-catenin signalling	0.0170
Complement system	0.0191
Histidine metabolism	0.0263
Sphingolipid metabolism	0.0389

**Table 4:** Immunohistochemical analysis of Ep-CAM expression

	**Ep-CAM expression**		
	**Negative**	**Positive**	***P*-value**
Responders	10	0	0.0528
Non-responders	14	6	

## References

[bib1] Barker N, Clevers H (2006) Mining the Wnt pathway for cancer therapeutics. Nat Rev Drug Discov 5: 997–1014 17139285 10.1038/nrd2154

[bib2] Baron S, Dianzani F (1994) The interferons: a biological system with therapeutic potential in viral infections. Antiviral Res 24: 97–110 7526796 10.1016/0166-3542(94)90058-2

[bib3] Branda M, Wands JR (2006) Signal transduction cascades and hepatitis B and C related hepatocellular carcinoma. Hepatology 43: 891–902 16628664 10.1002/hep.21196

[bib4] Breuhahn K, Baeuerle PA, Peters M, Prang N, Tox U, Kohne-Volland R, Dries V, Schirmacher P, Leo E (2006) Expression of epithelial cellular adhesion molecule (Ep-CAM) in chronic (necro-)inflammatory liver diseases and hepatocellular carcinoma. Hepatol Res 34: 50–56 16364680 10.1016/j.hepres.2005.10.006

[bib5] Calvano SE, Xiao W, Richards DR, Felciano RM, Baker HV, Cho RJ, Chen RO, Brownstein BH, Cobb JP, Tschoeke SK, Miller-Graziano C, Moldawer LL, Mindrinos MN, Davis RW, Tompkins RG, Lowry SF (2005) A network-based analysis of systemic inflammation in humans. Nature 437: 1032–1037 16136080 10.1038/nature03985

[bib6] Chang HW, Roh JL, Jeong EJ, Lee SW, Kim SW, Choi SH, Park SK, Kim SY (2008) Wnt signaling controls radiosensitivity via cyclooxygenase-2-mediated Ku expression in head and neck cancer. Int J Cancer 122: 100–107 17764107 10.1002/ijc.23069

[bib7] Damdinsuren B, Nagano H, Kondo M, Natsag J, Hanada H, Nakamura M, Wada H, Kato H, Marubashi S, Miyamoto A, Takeda Y, Umeshita K, Dono K, Monden M (2006) TGF-beta1-induced cell growth arrest and partial differentiation is related to the suppression of Id1 in human hepatoma cells. Oncol Rep 15: 401–408 16391861

[bib8] Damdinsuren B, Nagano H, Wada H, Noda T, Natsag J, Marubashi S, Miyamoto A, Takeda Y, Umeshita K, Doki Y, Dono K, Monden M (2007) Interferon alpha receptors are important for antiproliferative effect of interferon-alpha against human hepatocellular carcinoma cells. Hepatol Res 37: 77–83 17300701 10.1111/j.1872-034X.2007.00007.x

[bib9] de La Coste A, Romagnolo B, Billuart P, Renard CA, Buendia MA, Soubrane O, Fabre M, Chelly J, Beldjord C, Kahn A, Perret C (1998) Somatic mutations of the beta-catenin gene are frequent in mouse and human hepatocellular carcinomas. Proc Natl Acad Sci USA 95: 8847–8851 9671767 10.1073/pnas.95.15.8847 PMC21165

[bib10] Eguchi H, Nagano H, Yamamoto H, Miyamoto A, Kondo M, Dono K, Nakamori S, Umeshita K, Sakon M, Monden M (2000) Augmentation of antitumor activity of 5-fluorouracil by interferon alpha is associated with up-regulation of p27Kip1 in human hepatocellular carcinoma cells. Clin Cancer Res 6: 2881–2890 10914738

[bib11] Gastl G, Spizzo G, Obrist P, Dunser M, Mikuz G (2000) Ep-CAM overexpression in breast cancer as a predictor of survival. Lancet 356: 1981–1982 11130529 10.1016/S0140-6736(00)03312-2

[bib12] Giles Jr RC, Tramontin R, Kadel WL, Whitaker K, Miksch D, Bryant DW, Fayer R (1980) Sarcocystosis in cattle in Kentucky. J Am Vet Med Assoc 176: 543–548 6767674

[bib13] Gutterman JU (1994) Cytokine therapeutics: lessons from interferon alpha. Proc Natl Acad Sci USA 91: 1198–1205 8108387 10.1073/pnas.91.4.1198 PMC43124

[bib14] Herlyn M, Steplewski Z, Herlyn D, Koprowski H (1979) Colorectal carcinoma-specific antigen: detection by means of monoclonal antibodies. Proc Natl Acad Sci USA 76: 1438–1442 286328 10.1073/pnas.76.3.1438 PMC383267

[bib15] Jamieson CH, Ailles LE, Dylla SJ, Muijtjens M, Jones C, Zehnder JL, Gotlib J, Li K, Manz MG, Keating A, Sawyers CL, Weissman IL (2004) Granulocyte-macrophage progenitors as candidate leukemic stem cells in blast-crisis CML. N Engl J Med 351: 657–667 15306667 10.1056/NEJMoa040258

[bib16] Kittaka N, Takemasa I, Takeda Y, Marubashi S, Nagano H, Umeshita K, Dono K, Matsubara K, Matsuura N, Monden M (2008) Molecular mapping of human hepatocellular carcinoma provides deeper biological insight from genomic data. Eur J Cancer 44: 885–897 18337085 10.1016/j.ejca.2008.02.019

[bib17] Klaus A, Birchmeier W (2008) Wnt signalling and its impact on development and cancer. Nat Rev Cancer 8: 387–398 18432252 10.1038/nrc2389

[bib18] Komori T, Takemasa I, Yamasaki M, Motoori M, Kato T, Kikkawa N, Kawaguchi N, Ikeda M, Yamamoto H, Sekimoto M, Matsubara K, Matsuura N, Monden M (2008) Gene expression of colorectal cancer: Preoperative genetic diagnosis using endoscopic biopsies. Int J Oncol 32: 367–375 18202759

[bib19] Kondo M, Nagano H, Wada H, Damdinsuren B, Yamamoto H, Hiraoka N, Eguchi H, Miyamoto A, Yamamoto T, Ota H, Nakamura M, Marubashi S, Dono K, Umeshita K, Nakamori S, Sakon M, Monden M (2005) Combination of IFN-alpha and 5-fluorouracil induces apoptosis through IFN-alpha/beta receptor in human hepatocellular carcinoma cells. Clin Cancer Res 11: 1277–1286 15709199

[bib20] Kondo M, Yamamoto H, Nagano H, Okami J, Ito Y, Shimizu J, Eguchi H, Miyamoto A, Dono K, Umeshita K, Matsuura N, Wakasa K, Nakamori S, Sakon M, Monden M (1999) Increased expression of COX-2 in nontumor liver tissue is associated with shorter disease-free survival in patients with hepatocellular carcinoma. Clin Cancer Res 5: 4005–4012 10632332

[bib21] Kurokawa Y, Matoba R, Nagano H, Sakon M, Takemasa I, Nakamori S, Dono K, Umeshita K, Ueno N, Ishii S, Kato K, Monden M (2004a) Molecular prediction of response to 5-fluorouracil and interferon-alpha combination chemotherapy in advanced hepatocellular carcinoma. Clin Cancer Res 10: 6029–6038 15447987 10.1158/1078-0432.CCR-04-0243

[bib22] Kurokawa Y, Matoba R, Takemasa I, Nagano H, Dono K, Nakamori S, Umeshita K, Sakon M, Ueno N, Oba S, Ishii S, Kato K, Monden M (2004b) Molecular-based prediction of early recurrence in hepatocellular carcinoma. J Hepatol 41: 284–291 15288478 10.1016/j.jhep.2004.04.031

[bib23] Kusaba M, Nakao K, Goto T, Nishimura D, Kawashimo H, Shibata H, Motoyoshi Y, Taura N, Ichikawa T, Hamasaki K, Eguchi K (2007) Abrogation of constitutive STAT3 activity sensitizes human hepatoma cells to TRAIL-mediated apoptosis. J Hepatol 47: 546–555 17602782 10.1016/j.jhep.2007.04.017

[bib24] Litvinov SV, Velders MP, Bakker HA, Fleuren GJ, Warnaar SO (1994) Ep-CAM: a human epithelial antigen is a homophilic cell-cell adhesion molecule. J Cell Biol 125: 437–446 8163559 10.1083/jcb.125.2.437 PMC2120036

[bib25] Miyamoto A, Umeshita K, Sakon M, Nagano H, Eguchi H, Kishimoto S, Dono K, Nakamori S, Gotoh M, Monden M (2000) Advanced hepatocellular carcinoma with distant metastases, successfully treated by a combination therapy of alpha-interferon and oral tegafur/uracil. J Gastroenterol Hepatol 15: 1447–1451 11197059 10.1046/j.1440-1746.2000.02289.x

[bib26] Moon RT, Kohn AD, De Ferrari GV, Kaykas A (2004) WNT and beta-catenin signalling: diseases and therapies. Nat Rev Genet 5: 691–701 15372092 10.1038/nrg1427

[bib27] Motoori M, Takemasa I, Doki Y, Saito S, Miyata H, Takiguchi S, Fujiwara Y, Yasuda T, Yano M, Kurokawa Y, Komori T, Yamasaki M, Ueno N, Oba S, Ishii S, Monden M, Kato K (2006) Prediction of peritoneal metastasis in advanced gastric cancer by gene expression profiling of the primary site. Eur J Cancer 42: 1897–1903 16831544 10.1016/j.ejca.2006.04.007

[bib28] Motoori M, Takemasa I, Yano M, Saito S, Miyata H, Takiguchi S, Fujiwara Y, Yasuda T, Doki Y, Kurokawa Y, Ueno N, Oba S, Ishii S, Monden M, Kato K (2005) Prediction of recurrence in advanced gastric cancer patients after curative resection by gene expression profiling. Int J Cancer 114: 963–968 15645432 10.1002/ijc.20808

[bib29] Nagano H, Miyamoto A, Wada H, Ota H, Marubashi S, Takeda Y, Dono K, Umeshita K, Sakon M, Monden M (2007a) Interferon-alpha and 5-fluorouracil combination therapy after palliative hepatic resection in patients with advanced hepatocellular carcinoma, portal venous tumor thrombus in the major trunk, and multiple nodules. Cancer 110: 2493–2501 17941012 10.1002/cncr.23033

[bib30] Nagano H, Sakon M, Eguchi H, Kondo M, Yamamoto T, Ota H, Nakamura M, Wada H, Damdinsuren B, Marubashi S, Miyamoto A, Takeda Y, Dono K, Umeshit K, Nakamori S, Monden M (2007b) Hepatic resection followed by IFN-alpha and 5-FU for advanced hepatocellular carcinoma with tumor thrombus in the major portal branch. Hepatogastroenterology 54: 172–179 17419255

[bib31] Nakamura M, Nagano H, Sakon M, Yamamoto T, Ota H, Wada H, Damdinsuren B, Noda T, Marubashi S, Miyamoto A, Takeda Y, Umeshita K, Nakamori S, Dono K, Monden M (2007) Role of the Fas/FasL pathway in combination therapy with interferon-alpha and fluorouracil against hepatocellular carcinoma *in vitro*. J Hepatol 46: 77–88 17045692 10.1016/j.jhep.2006.07.032

[bib32] Obi S, Yoshida H, Toune R, Unuma T, Kanda M, Sato S, Tateishi R, Teratani T, Shiina S, Omata M (2006) Combination therapy of intraarterial 5-fluorouracil and systemic interferon-alpha for advanced hepatocellular carcinoma with portal venous invasion. Cancer 106: 1990–1997 16565970 10.1002/cncr.21832

[bib33] Ogawa M, Yamamoto H, Nagano H, Miyake Y, Sugita Y, Hata T, Kim BN, Ngan CY, Damdinsuren B, Ikenaga M, Ikeda M, Ohue M, Nakamori S, Sekimoto M, Sakon M, Matsuura N, Monden M (2004) Hepatic expression of ANG2 RNA in metastatic colorectal cancer. Hepatology 39: 528–539 14768007 10.1002/hep.20048

[bib34] Ohigashi T, Mizuno R, Nakashima J, Marumo K, Murai M (2005) Inhibition of Wnt signaling downregulates Akt activity and induces chemosensitivity in PTEN-mutated prostate cancer cells. Prostate 62: 61–68 15389810 10.1002/pros.20117

[bib35] Oken MM, Creech RH, Tormey DC, Horton J, Davis TE, McFadden ET, Carbone PP (1982) Toxicity and response criteria of the Eastern Cooperative Oncology Group. Am J Clin Oncol 5: 649–655 7165009

[bib36] Ota H, Nagano H, Sakon M, Eguchi H, Kondo M, Yamamoto T, Nakamura M, Damdinsuren B, Wada H, Marubashi S, Miyamoto A, Dono K, Umeshita K, Nakamori S, Wakasa K, Monden M (2005) Treatment of hepatocellular carcinoma with major portal vein thrombosis by combined therapy with subcutaneous interferon-alpha and intra-arterial 5-fluorouracil; role of type 1 interferon receptor expression. Br J Cancer 93: 557–564 16106266 10.1038/sj.bjc.6602742 PMC2361594

[bib37] Patt YZ, Yoffe B, Charnsangavej C, Pazdur R, Fischer H, Cleary K, Roh M, Smith R, Noonan CA, Levin B (1993) Low serum alpha-fetoprotein level in patients with hepatocellular carcinoma as a predictor of response to 5-FU and interferon-alpha-2b. Cancer 72: 2574–2582 7691392 10.1002/1097-0142(19931101)72:9<2574::aid-cncr2820720911>3.0.co;2-l

[bib38] Reya T, Clevers H (2005) Wnt signalling in stem cells and cancer. Nature 434: 843–850 15829953 10.1038/nature03319

[bib39] Rhodes DR, Chinnaiyan AM (2005) Integrative analysis of the cancer transcriptome. Nat Genet 37(Suppl): S31–S37 15920528 10.1038/ng1570

[bib40] Sakon M, Nagano H, Dono K, Nakamori S, Umeshita K, Yamada A, Kawata S, Imai Y, Iijima S, Monden M (2002) Combined intraarterial 5-fluorouracil and subcutaneous interferon-alpha therapy for advanced hepatocellular carcinoma with tumor thrombi in the major portal branches. Cancer 94: 435–442 11900229 10.1002/cncr.10246

[bib41] Sato N, Meijer L, Skaltsounis L, Greengard P, Brivanlou AH (2004) Maintenance of pluripotency in human and mouse embryonic stem cells through activation of Wnt signaling by a pharmacological GSK-3-specific inhibitor. Nat Med 10: 55–63 14702635 10.1038/nm979

[bib42] Wada H, Nagano H, Yamamoto H, Arai I, Ota H, Nakamura M, Damdinsuren B, Noda T, Marubashi S, Miyamoto A, Takeda Y, Umeshita K, Doki Y, Dono K, Nakamori S, Sakon M, Monden M (2007) Combination therapy of interferon-alpha and 5-fluorouracil inhibits tumor angiogenesis in human hepatocellular carcinoma cells by regulating vascular endothelial growth factor and angiopoietins. Oncol Rep 18: 801–809 17786339

[bib43] Woodward WA, Chen MS, Behbod F, Alfaro MP, Buchholz TA, Rosen JM (2007) WNT/beta-catenin mediates radiation resistance of mouse mammary progenitor cells. Proc Natl Acad Sci USA 104: 618–623 17202265 10.1073/pnas.0606599104 PMC1766434

[bib44] Yamamoto T, Nagano H, Sakon M, Wada H, Eguchi H, Kondo M, Damdinsuren B, Ota H, Nakamura M, Wada H, Marubashi S, Miyamoto A, Dono K, Umeshita K, Nakamori S, Yagita H, Monden M (2004) Partial contribution of tumor necrosis factor-related apoptosis-inducing ligand (TRAIL)/TRAIL receptor pathway to antitumor effects of interferon-alpha/5-fluorouracil against Hepatocellular Carcinoma. Clin Cancer Res 10: 7884–7895 15585621 10.1158/1078-0432.CCR-04-0794

[bib45] Yamashina K, Yamamoto H, Chayama K, Nakajima K, Kikuchi A (2006) Suppression of STAT3 activity by Duplin, which is a negative regulator of the Wnt signal. J Biochem 139: 305–314 16452319 10.1093/jb/mvj033

[bib46] Yamashita T, Budhu A, Forgues M, Wang XW (2007) Activation of hepatic stem cell marker EpCAM by Wnt-beta-catenin signaling in hepatocellular carcinoma. Cancer Res 67: 10831–10839 18006828 10.1158/0008-5472.CAN-07-0908

[bib47] Yang W, Yan HX, Chen L, Liu Q, He YQ, Yu LX, Zhang SH, Huang DD, Tang L, Kong XN, Chen C, Liu SQ, Wu MC, Wang HY (2008) Wnt/beta-catenin signaling contributes to activation of normal and tumorigenic liver progenitor cells. Cancer Res 68: 4287–4295 18519688 10.1158/0008-5472.CAN-07-6691

[bib48] Yang XJ, Tan MH, Kim HL, Ditlev JA, Betten MW, Png CE, Kort EJ, Futami K, Furge KA, Takahashi M, Kanayama HO, Tan PH, Teh BS, Luan C, Wang K, Pins M, Tretiakova M, Anema J, Kahnoski R, Nicol T, Stadler W, Vogelzang NG, Amato R, Seligson D, Figlin R, Belldegrun A, Rogers CG, Teh BT (2005) A molecular classification of papillary renal cell carcinoma. Cancer Res 65: 5628–5637 15994935 10.1158/0008-5472.CAN-05-0533

[bib49] Zembutsu H, Ohnishi Y, Tsunoda T, Furukawa Y, Katagiri T, Ueyama Y, Tamaoki N, Nomura T, Kitahara O, Yanagawa R, Hirata K, Nakamura Y (2002) Genome-wide cDNA microarray screening to correlate gene expression profiles with sensitivity of 85 human cancer xenografts to anticancer drugs. Cancer Res 62: 518–527 11809704

